# Cardiovascular disease risk factors and infertility: multivariable analyses and one-sample Mendelian randomization analyses in the Trøndelag Health Study

**DOI:** 10.1093/hropen/hoae033

**Published:** 2024-05-24

**Authors:** Karoline H Skåra, Álvaro Hernáez, Øyvind Næss, Abigail Fraser, Deborah A Lawlor, Stephen Burgess, Ben M Brumpton, Maria C Magnus

**Affiliations:** Centre for Fertility and Health, Norwegian Institute of Public Health, Oslo, Norway; Department of Community Medicine and Global Health, Institute of Health and Society, University of Oslo, Oslo, Norway; Centre for Fertility and Health, Norwegian Institute of Public Health, Oslo, Norway; Consorcio CIBER, M.P. Enfermedades Cardiovasculares (CIBERCV), Instituto de Salud Carlos III, Madrid, Spain; Blanquerna School of Health Sciences, Universitat Ramon Llull, Barcelona, Spain; Department of Community Medicine and Global Health, Institute of Health and Society, University of Oslo, Oslo, Norway; Division of Mental and Physical Health, Norwegian Institute of Public Health, Oslo, Norway; MRC Integrative Epidemiology Unit, University of Bristol, Bristol, UK; Population Health Sciences, Bristol Medical School, University of Bristol, Bristol, UK; MRC Integrative Epidemiology Unit, University of Bristol, Bristol, UK; Population Health Sciences, Bristol Medical School, University of Bristol, Bristol, UK; MRC Biostatistics Unit, University of Cambridge, Cambridge, UK; Cardiovascular Epidemiology Unit, Department of Public Health and Primary Care, University of Cambridge, Cambridge, UK; HUNT Center for Molecular and Clinical Epidemiology, Department of Public Health and Nursing, NTNU Norwegian University of Science and Technology, Levanger, Norway; HUNT Research Centre, Department of Public Health and Nursing, NTNU Norwegian University of Science and Technology, Levanger, Norway; Clinic of Medicine, St Olavs Hospital, Trondheim University, Trondheim, Norway; Centre for Fertility and Health, Norwegian Institute of Public Health, Oslo, Norway

**Keywords:** infertility, female infertility, male infertility, childless, cardiovascular disease, risk factors, the HUNT Study, the Trøndelag Health Study, Mendelian randomization

## Abstract

**STUDY QUESTION:**

Are cardiovascular disease (CVD) risk factors causally associated with higher risk of infertility among women and men?

**SUMMARY ANSWER:**

We found evidence to support a causal relationship between smoking initiation and history of infertility in women.

**WHAT IS KNOWN ALREADY:**

Several CVD risk factors are associated with history of infertility. Previous studies using Mendelian randomization (MR) further support a causal relationship between BMI and infertility in women.

**STUDY DESIGN, SIZE, DURATION:**

We used data from the Trøndelag Health Study (HUNT) in Norway, a prospective population-based cohort study, including 26 811 women and 15 598 men participating in three survey collections in 1995–1997 (HUNT2), 2006–2008 (HUNT3), and 2017–2019 (HUNT4).

**PARTICIPANTS/MATERIALS, SETTING, METHODS:**

Our outcome was women’s self-reported history of infertility, defined as ever having tried to conceive for 12 months or more or having used ART. We assigned the history of infertility reported by women to their male partners; therefore, the measure of infertility was on the couple level. We used both conventional multivariable analyses and one-sample MR analyses to evaluate the association between female and male CVD risk factors (including BMI, blood pressure, lipid profile measurements, and smoking behaviours) and history of infertility in women and men, separately.

**MAIN RESULTS AND THE ROLE OF CHANCE:**

A total of 4702 women (18%) and 2508 men (16%) were classified with a history of infertility. We found a higher risk of infertility among female smokers compared to non-smokers in both multivariable and MR analyses (odds ratio (OR) in multivariable analysis, 1.20; 95% CI, 1.12–1.28; OR in MR analysis, 1.13; CI, 1.02–1.26), and potentially for higher BMI (OR in multivariable analysis, 1.13; CI, 1.09–1.18; OR in MR analysis, 1.11, CI, 0.92–1.34). In multivariable analysis in women, we also found evidence of associations between triglyceride levels, high-density lipoprotein cholesterol, lifetime smoking index, and smoking intensity with higher risk of infertility. However, these results were not consistent in MR analyses. We found no robust or consistent associations between male CVD risk factors and infertility.

**LIMITATIONS, REASONS FOR CAUTION:**

Our main limitation was that the CVD risk factors measured might not adequately capture the relevant time periods for when couples were trying to conceive. Additionally, we did not have information on causes of infertility in either women or men.

**WIDER IMPLICATIONS OF THE FINDINGS:**

Women with infertility could have a worse CVD risk factor profile and thus public health interventions aimed at reducing the impact of some CVD risk factors, such as smoking and BMI, could reduce the burden of infertility. However, additional MR studies of the relationship between CVD risk factors and infertility with a larger sample size would be of value.

**STUDY FUNDING/COMPETING INTEREST(S):**

The study was supported by a grant from the European Research Council under the European Union’s Horizon 2020 research and innovation program (grant agreements no. 947684). This research was also supported by the Research Council of Norway through its Centres of Excellence funding scheme (project no. 262700) and partly funded by the Research Council of Norway, project: Women’s fertility—an essential component of health and well-being (project no. 320656). D.A.L. and A.F. work in a unit that is supported by the University of Bristol and the UK Medical Research Council (MC_UU_00011/6). D.A.L.’s contribution to the article is supported by the European Research Council (101021566), the British Heart Foundation (CH/F/20/90003 and AA/18/7/34219). S.B.’s contribution to the article is supported by the Wellcome Trust (225790/Z/22/Z). B.M.B. is funded by The Liaison Committee for education, research and innovation in Central Norway; and the Joint Research Committee between St. Olavs Hospital and the Faculty of Medicine and Health Sciences, NTNU. The genotyping in HUNT was financed by the National Institute of Health (NIH); University of Michigan; The Research Council of Norway; The Liaison Committee for education, research and innovation in Central Norway; and the Joint Research Committee between St. Olavs Hospital and the Faculty of Medicine and Health Sciences, NTNU. None of the funding organizations influenced the study design, reporting, or interpretation of results. The views expressed in the present article are those of the authors and not necessarily any acknowledged funding organization. D.A.L. reports grants from Medtronic Ltd and Roche Diagnostics outside the submitted work. The other authors have no conflicts of interest.

**TRIAL REGISTRATION NUMBER:**

N/A.

WHAT DOES THIS MEAN FOR PATIENTS? Infertility, defined as being unable to become pregnant after unprotected intercourse for 1 year, is poorly understood in many cases. Some conditions are known to contribute to infertility, such as polycystic ovary syndrome and endometriosis in women, but it remains unexplained in a large proportion of cases. This study looked at a large group of women and men from the Trøndelag Health Study in Norway who reported whether they had ever tried to get pregnant for more than a year, that is, had a history of infertility. The researchers wanted to see if the women and men who had higher BMI, blood pressure, levels of cholesterol, or were smokers, had a higher risk of having experienced infertility. They only found a relationship between smoking and infertility, and potentially between BMI and infertility, among women. Additional studies are needed, particularly studies with detailed information on the underlying contributing causes of infertility, including whether it reflects male factors, female factors, or a combination of both.

## Introduction

Fertility rates are decreasing worldwide, but the decline is most pronounced in high-income countries (Murray *et al.*, 2018). Reproductive choices, such as delayed childbearing, are most likely important drivers, but other potential modifiable risk factors contributing to infertility may also exist (Crawford and Steiner, 2015). Infertility, defined as being unable to establish a clinical pregnancy after 1 year of trying, affects 10–17% of couples (Agarwal *et al.*, 2015; Zegers-Hochschild *et al.*, 2017; Skåra *et al.*, 2022). Evidence supports a potential increased risk of cardiovascular disease (CVD) among women with a history of infertility (Parikh *et al.*, 2012; Gleason *et al.*, 2019; Magnus *et al.*, 2021; Murugappan *et al.*, 2022; Skåra *et al.*, 2022). It is therefore important to understand whether this observation might at least partly reflect an underlying relationship between classic CVD risk factors and infertility. The potentially modifiable nature of these CVD risk factors also opens the possibility to explore interventions to reduce the burden of infertility.

Studies have found that high blood pressure is associated with a higher risk of uterine fibroids, which are linked to menstrual irregularities and infertility in women (Newbold *et al.*, 2000; Faerstein *et al.*, 2001; Boynton-Jarrett *et al.*, 2005). Studies have also shown that high BMI is associated with infertility among both women and men (Best *et al.*, 2017; Mahalingaiah *et al.*, 2017; Hernáez *et al.*, 2021; Venkatesh *et al.*, 2022), although intervention trials have failed to find an association (Mutsaerts *et al.*, 2016; Einarsson *et al.*, 2017). More robust evidence is found for a role of smoking in infertility among women (Waylen *et al.*, 2009; Radin *et al.*, 2014; Hyland *et al.*, 2015; Yuan *et al.*, 2021).

Whether the associations between CVD risk factors and infertility reflect a causal effect remains uncertain (Smith and Ebrahim, 2004). For example, there are known socio-demographic variations in the risk factors of interest, which could result in a complicated confounding pattern, which is difficult to fully account for (Oh *et al.*, 2010; Dathan-Stumpf *et al.*, 2016; Marques *et al.*, 2017). There is also the possibility that individuals who experienced infertility might have changed their behaviours as part of their attempt to conceive, resulting in reverse causation (Hanevik *et al.*, 2021). Randomized controlled trials (RCTs) overcome reverse causation and unobserved confounding by design. However, setting up large and robust RCTs is expensive and time-consuming (Lee and Lim, 2019). Mendelian randomization (MR) mimics RCTs by using genetic variants associated with the exposure of interest as instrumental variables to explore unconfounded effects, based on the fact that they are randomly allocated at conception (Davey Smith and Ebrahim, 2003; Lee and Lim, 2019). In our previous work in the Norwegian Mother, Father, and Child Cohort (MoBa), MR analyses supported a causal relationship between BMI, but not smoking, with infertility (Hernáez *et al.*, 2021, 2022).

The objective of our study was to explore the causal nature of the relationship between established CVD risk factors and infertility among women and men by comparing results from conventional multivariable analyses with one-sample MR analyses. We expand on previous findings by investigating the associations in a large population-based cohort in Norway, including couples who never conceived, and exploring a broader range of CVD risk factors.

## Materials and methods

### Study population

We used data from the Trøndelag Health (HUNT) Study, a prospective population-based cohort study with data on general health, biological samples, and anthropometric measurements, collected through questionnaires and clinical examinations. We included women and men who participated in at least one of three waves of data collection in HUNT: the HUNT2 Survey (1995–1997, 65 237 people), the HUNT3 Survey (2006–2008, 50 807 people), and/or the HUNT4 Survey (2017–2019, 56 042 people) (Krokstad *et al.*, 2013). The HUNT1 Survey was also carried out in 1984–1986, but because this survey did not include a question regarding infertility, we did not have access to data from the HUNT1 Survey (Krokstad *et al.*, 2013; Åsvold *et al.*, 2023).

**Figure 1.**
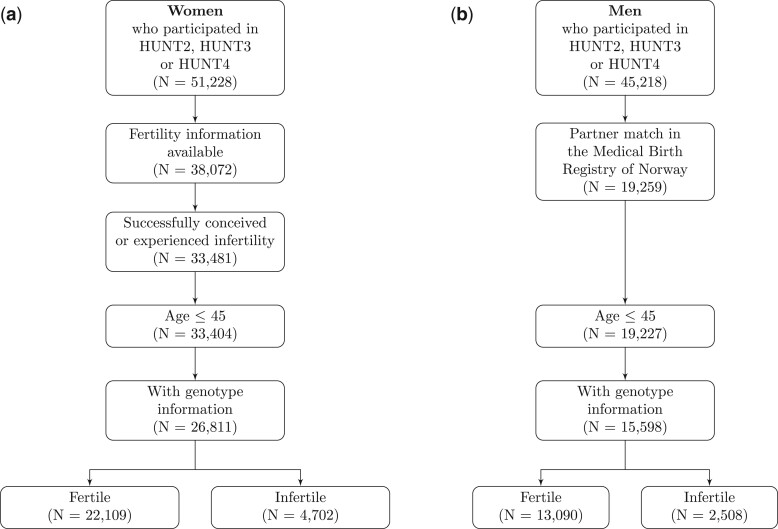
**Illustration of the eligible population in a study of cardiovascular disease risk factors and infertility.** Study populations for (**a**) women and (**b**) men are shown. HUNT, Trøndelag Health Study.

We linked self-reported information with the Medical Birth Registry of Norway using unique national identification numbers. We included participants with information on infertility history, who were not voluntarily childless (voluntarily childless defined as not having a history of infertility and no self-reported or registered pregnancy in the birth registry), who had been genotyped, who had measurements of CVD risk factors at 45 years or younger, and who had measurements of CVD risk factors prior to or at the same time as when information on history of infertility was obtained (Fig. 1); the latter was done to reduce the risk of reverse causation. The following work is described according to the Strengthening the Reporting of Observational Studies in Epidemiology (STROBE) guidelines for reporting MR and cohort studies. The present study was approved by the Regional Committee for Medical and Health Research Ethics of South/East Norway (REK 2017/78545). Written informed consent was obtained from all participants.

### CVD risk factors

The CVD risk factors included BMI (weight in kg divided by squared height in m), systolic and diastolic blood pressure (SBP and DBP, respectively; mmHg), high-density lipoprotein cholesterol (HDL cholesterol; mg/dl), triglycerides (log mg/dl), low-density lipoprotein cholesterol (LDL cholesterol; mg/dl), smoking initiation (yes/no), smoking intensity for former and current daily smokers (number of cigarettes per day), and lifetime smoking index (Wootton *et al.*, 2020). Measures of metabolic factors were taken by trained health personnel during clinical examinations. Lipid levels were determined using an enzymatic colorimetric method in non-fasting serum (Lindemann *et al.*, 2009). LDL cholesterol was calculated using non-HDL cholesterol and triglycerides using the equation developed by Sampson *et al.* (2020). We used the earliest recorded measurement for each CVD risk factor for individuals participating in multiple HUNT surveys. Smoking information was collected from all surveys to capture lifetime smoking history. Lifetime smoking index was calculated considering smoking duration, heaviness, and cessation for both daily and occasional smokers and the following formula: $(1-0.5^{\frac{\mathrm{dur}}{\tau }}){(0.5}^{\frac{\mathrm{tsc}}{\tau }})\ln\left(\mathrm{int}+1\right)$ where *dur* is the duration of smoking, *τ* is the half-life, *tsc* is the time since cessation, and *int* is the number of cigarettes per day (Wootton *et al.*, 2020). Half-life represents the exponentially decreasing effect of smoking on the outcome, for which we used a value of 18 based on the simulations conducted by Wootton *et al.* (2020). Individuals with no smoking exposure will have a value of zero.

### Genetic predictors of CVD risk factors for use in one-sample MR analyses

Genotyping details in HUNT can be found elsewhere (Nielsen *et al.*, 2018; Brumpton *et al.*, 2020, 2022). We selected independent single-nucleotide polymorphisms (SNPs) significantly associated with each CVD risk factor of interest (*P* < 5 × 10^−8^) based on the most recent genome-wide association studies (GWASs). The alleles were ordered so that a higher allele dosage (ranging from 0 to 2) reflected increasing levels of CVD risk factors. We calculated genetic risk scores (GRSs) by summing the weighted risk alleles using effect sizes from the GWASs and the following formula: $\mathrm{GRS}=\sum_{i=1}^{m}\beta_{i}{\mathrm{SNP}}_{i}$, where $\beta_{i}$ is the effect of the ith SNP for the given CVD risk factor, and ${\mathrm{SNP}}_{i}$ is the dosage of the effect allele of the ith SNP. GRSs were weighted by dividing each GRS by the total SNP effect on the exposure of interest and multiplying by the total number of SNPs. The number of SNPs included in each GRS, ranging from 55 for smoking intensity to 939 for BMI, and the source GWASs are indicated in Supplementary Table S1.

### Infertility

Women were asked about their lifetime history of infertility through the question: ‘Have you ever tried for more than a year to become pregnant?’ Those who answered ‘Yes’ in any survey to this question, or who had a registered pregnancy in the birth registry resulting from ART, were classified as infertile. Men were not asked about their history of infertility, so we identified those male partners of women who also participated in HUNT by linkage to the Medical Birth Registry of Norway, as previously described, and assigned them the response of their partners (Skåra *et al.*, 2022). The measure of infertility was therefore on the couple level. For women with a history of infertility, we further classified them according to whether they were parous (at least one self-reported or registered birth) or not. Because men were identified through the Medical Birth Registry of Norway, this limited us to only including parous men in our study population.

### Statistical analyses

We estimated the association between a one SD increase in continuous CVD risk factors and the risk of infertility by logistic regression, using the SD of the measured CVD risk factors to scale both the measured and the genetically predicted CVD risk factors. This was done to ensure comparability between multivariable and MR analyses. For smoking initiation, we examined the difference in the odds of infertility between smokers and non-smokers. The multivariable analyses adjusted for age at report of infertility history (continuous) and education level (categorical; higher education, upper secondary school, and secondary school). In one-sample MR, we used the GRSs to calculate genetically predicted CVD risk factor effects on infertility using two-stage least square (2SLS) regression, adjusting for the first 10 ancestry-informative genetic principal components and genotype batch (Palmer *et al.*, 2011).

We also explored potential non-linear relationships between CVD risk factors and infertility. In the multivariable analyses, we employed generalized additive models with restricted cubic splines using the *mgcv* R package, and evaluated the model fit based on the effective degrees of freedom and the Akaike information criterion (Wood, 2017). In the MR analyses, we initially planned to also explore non-linear MR effects but because of recent concerns about potential biases in non-linear MR and ongoing debates about the methods, we did not do so (Hamilton *et al.*, 2024; Burgess, 2024).

The MR methods can be influenced by confounding owing to weak genetic instruments, population structure, or other factors that connect the genetic variants to the outcomes beyond the exposure of interest such as horizontal pleiotropy (Davey Smith and Ebrahim, 2003; Hemani *et al.*, 2018). Regarding potential weak instrument bias, we checked the strength of the association between the genetic instruments and their phenotypes directly in HUNT. Potential confounding owing to population structure was accounted for by adjusting for genomic principal components. To assess horizontal pleiotropy, we performed a sensitivity analysis using summary-level MR methods, by comparing the standard inverse variance weighted analysis (IVW) to the MR–Egger and weighted median approaches. IVW assumes no unbalanced horizontal pleiotropy as it forces the regression line through SNP-exposure and SNP-outcome coordinates to go through 0. MR-Egger does not make this assumption and it does not force the line through 0. The weighted median analysis is valid if <50% of the weight comes from SNPs that are not related to other risk factors for the outcome. Concordance in the estimates across these approaches reduces the concern regarding unbalanced horizontal pleiotropy (Hemani *et al.*, 2018).

For the multivariable analyses, we conducted a sensitivity analysis including participants without genotype data available, to evaluate potential selection bias caused by the restriction to individuals with genotype data. Other sensitivity analyses for both multivariable and MR analyses included excluding participants with ART pregnancies, as this is a group that might modify their behaviour as part of their treatment, and a sensitivity analysis restricting the study population to those without pregnancies prior to collection of information on the CVD risk factors, to minimize the possibility of reverse causation. As an additional subgroup analysis, we examined the risk of infertility among nulliparous women (no registered pregnancies) and parous women (at least one registered pregnancy), separately.

### Software

Analyses were performed in R software version 4.0.3 (RStudio Team, 2020; R Core Team, 2021).

## Results

The study population included 26 811 women (18% with history of infertility) and 15 598 men (16% with history of infertility) (Fig. 1). The median age at first experience of infertility among women with history of infertility was 25.6 (4.8) years. Both women and men with a history of infertility were similar in age at report of infertility compared to those without infertility (Table 1). The age at which the CVD risk factors were obtained was also similar among those with and without infertility for both women and men (Supplementary Table S2). Notably, the majority of individuals had their information on the CVD risk factors and the history of infertility from the same data collection (86%). Individuals with a history of infertility had fewer children and higher education (Table 1). The distribution of the CVD risk factors of interest according to history of infertility are shown in Supplementary Table S3.

**Table 1.** Distribution of background characteristics for the eligible study population.

Characteristics	Fertile women	Infertile women	Fertile men	Infertile men
Count, No.	22 109	4702	13 090	2508
Age at report of fertility history, years, median (IQR)	46.0 (35.6, 57.0)	41.8 (34.9, 51.1)	46.5 (37.8, 55.0)	43.7 (36.9, 51.5)
Cohabitation, No. (%)				
Living with cohabitant	18 889 (85.4)	4151 (88.3)	12 054 (92.1)	2296 (91.5)
Living without cohabitant	3220 (14.6)	551 (11.7)	1036 (7.9)	212 (8.5)
Missing	57 (0.3)	37 (0.8)	526 (4.0)	99 (3.9)
Education, No. (%)				
Higher education	6863 (31.0)	1693 (36.0)	4057 (31.0)	847 (33.8)
Upper secondary school	2553 (11.5)	662 (14.1)	1389 (10.6)	317 (12.6)
Secondary school	12 559 (56.8)	2318 (49.3)	7595 (58.0)	1336 (53.3)
Missing	134 (0.6)	29 (0.6)	49 (0.4)	8 (0.3)
Children, No. (%)				
0	0 (0.0)	594 (12.6)	0 (0.0)	24 (1.0)
1	1908 (8.6)	1001 (21.3)	772 (5.9)	535 (21.3)
2	8646 (39.1)	1653 (35.2)	5345 (40.8)	1078 (43.0)
≥3	11 555 (52.3)	1373 (29.2)	6973 (53.3)	879 (34.7)
Missing	0 (0.0)	81 (1.7)	0 (0.0)	0 (0.0)
Age (years) at first pregnancy, median (IQR)	23 (21, 27)	25 (22, 29)	27 (24, 30)	28 (25, 33)
Age (years) at first pregnancy, No. (%)				
19–29	19 078 (86.3)	3075 (65.4)	9131 (69.8)	1418 (56.5)
30–39	2621 (11.9)	927 (19.7)	3374 (25.8)	944 (37.6)
40–45	155 (0.7)	48 (1.0)	424 (3.2)	96 (3.8)
>45	7 (0.0)	2 (0.0)	124 (0.9)	38 (1.5)
Missing	239 (1.1)	649 (13.8)	0 (0.0)	0 (0.0)
Age (years) at first experienced infertility, No. (%)^a^				
19–29	NA	3497 (74.4)	NA	NA
30–39	NA	883 (18.8)	NA	NA
>39	NA	37 (0.8)	NA	NA
Missing	NA	285 (6.1)	NA	NA
ART, No. (%)				
No	22 109 (100.0)	4275 (90.9)	13 090 (100.0)	2229 (88.9)
Yes	0 (0.0)	427 (9.1)	0 (0.0)	279 (11.1)

a Data on age at first experienced infertility was not available (NA) for fertile women/all men because they were not infertile/asked about infertility.

### Multivariable analyses

**Figure 2.**
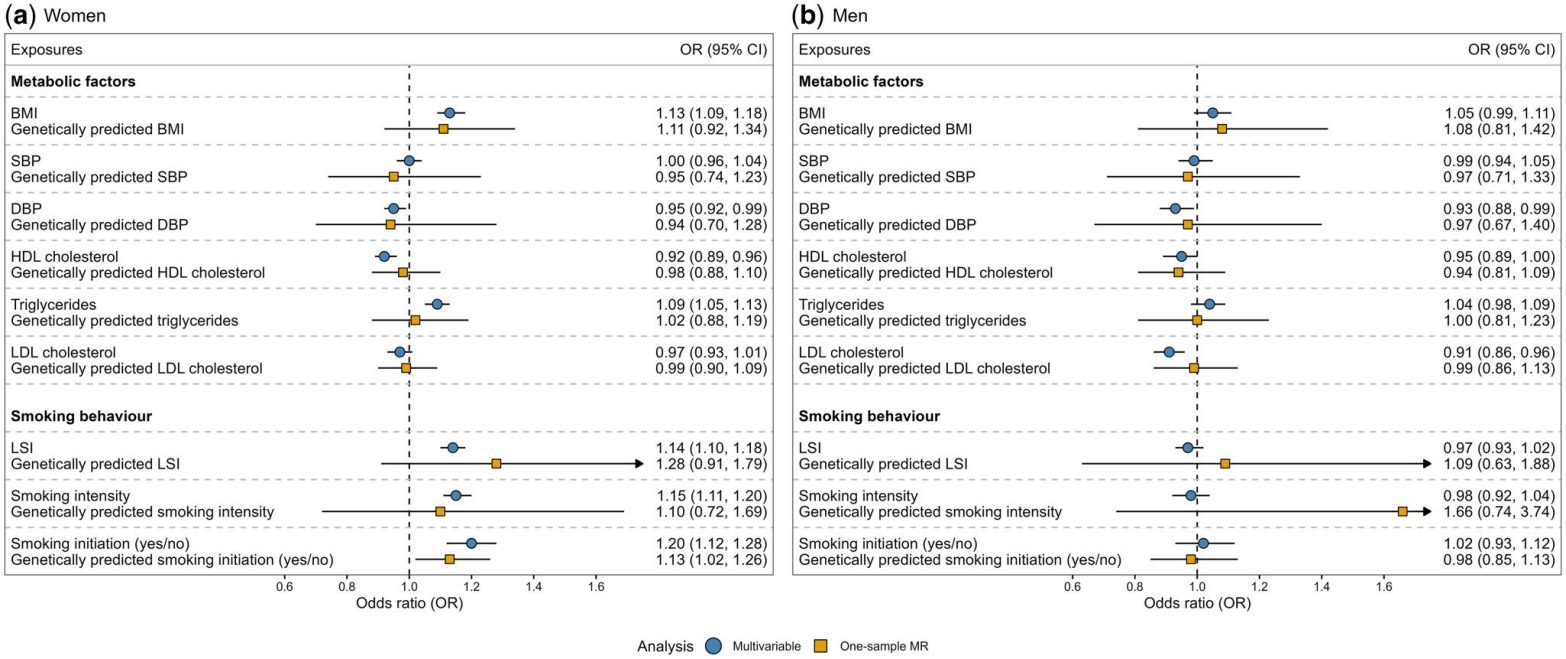
**Associations between cardiovascular disease risk factors and infertility**. Associations between one SD increase in the continuous CVD risk factors and the risk of infertility are shown for (**a**) women and (**b**) men in conventional multivariable analyses (blue circles) and one-sample MR analyses (orange squares). For smoking initiation, we examined the difference in the odds of infertility between smokers and non-smokers. DBP, diastolic blood pressure; CVD, cardiovascular disease; HDL, high-density lipoprotein; LDL, low-density lipoprotein; LSI, lifetime smoking index; MR, Mendelian randomization; SBP, systolic blood pressure.

In linear multivariable regression analyses among women, we found a higher risk of infertility with a higher BMI (odds ratio (OR), 1.13 per SD increase; CI, 1.09–1.18), higher levels of triglycerides (OR, 1.09 per SD increase; CI, 1.05–1.13), lifetime smoking index (OR, 1.14 per SD increase; CI, 1.10–1.18), smoking intensity (OR, 1.15 per SD increase; CI, 1.11–1.20), and smoking initiation (OR, 1.20; CI, 1.12–1.28). We also found a lower risk of infertility with higher levels of HDL cholesterol (OR, 0.92 per SD increase; CI, 0.89–0.96; Fig. 2). In men, we found a reduced risk of infertility with higher DBP (OR, 0.93 per SD increase; CI, 0.88–0.99) and LDL cholesterol (OR, 0.91 per SD increase; CI, 0.86–0.96; Fig. 2). No other relationships were observed among men.

**Figure 3.**
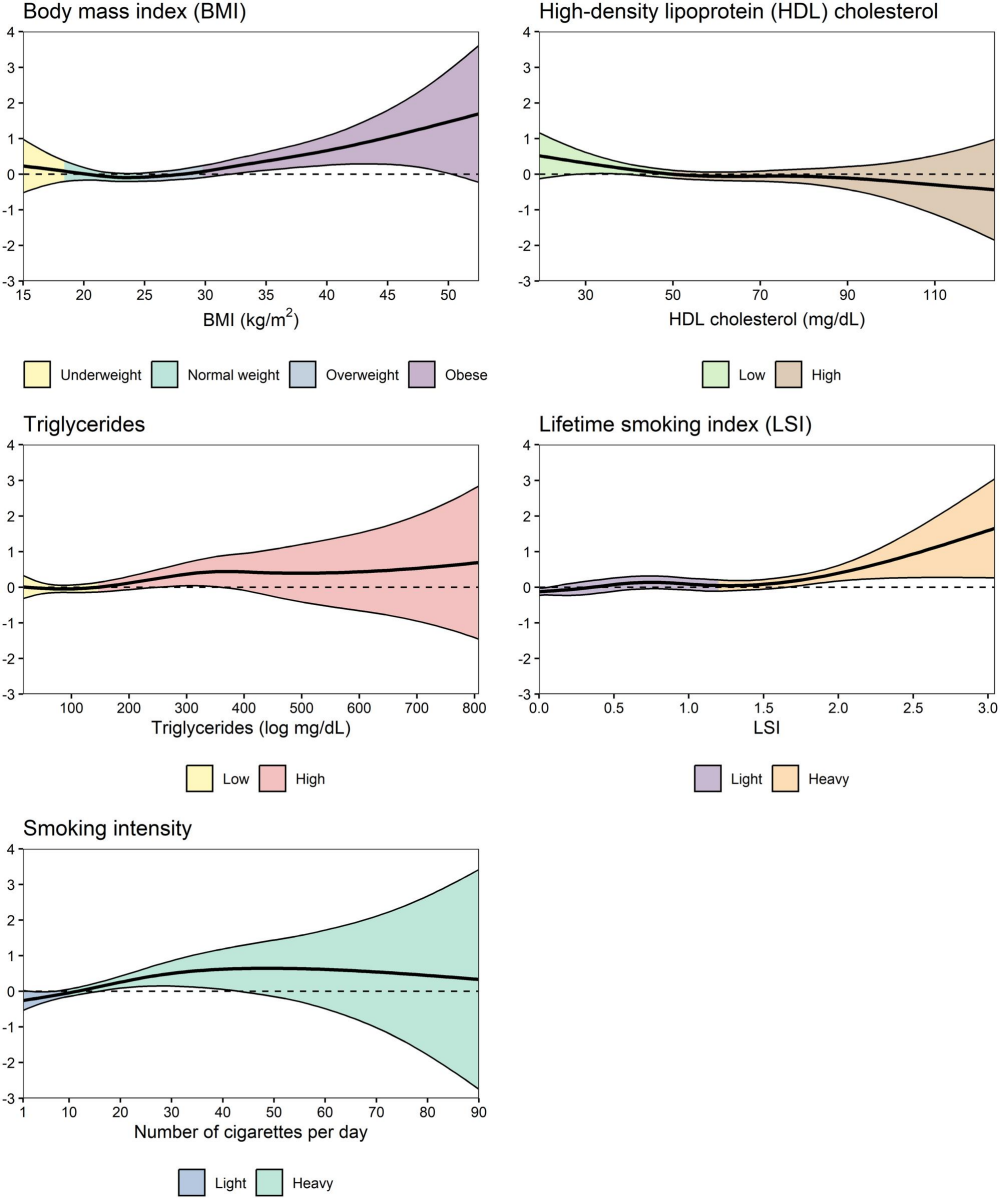
**Non-linear associations between one SD increase in continuous CVD risk factors and the risk of infertility for women in conventional multivariable analyses**. CIs are shaded according to clinical cut-offs for BMI (<25.0, 25.0–29.9, and ≥30 kg/m^2^) and lipid measures (HDL cholesterol, <50 and ≥50 mg/dl; triglycerides, <150 and ≥150 mg/dl), while median values were used for LSI (<1.2 and ≥1.2) and smoking intensity (<10 and ≥10 cigarettes per day). CVD, cardiovascular disease; HDL, high-density lipoprotein; LSI, lifetime smoking index.

For women, there was some evidence for a non-linear relationship with infertility in conventional multivariable analyses for BMI, HDL cholesterol, triglycerides, lifetime smoking index, and smoking intensity (Fig. 3; Supplementary Table S4). No potential non-linear associations were observed for men (Supplementary Table S4).

### One-sample MR analyses

All genetic instruments showed a strong predictive power among both men and women (*R*^2^≥1.8), with the exception for lifetime smoking index and smoking intensity, which showed a more modest predictive power (*R*^2^ ranging between 0.5 and 1.0; Supplementary Table S5). In our linear MR analyses, the only significant association was a higher risk of infertility with smoking initiation among women (OR, 1.13; CI, 1.02–1.26). The effect estimate for genetically predicted BMI among women was similar as for the conventional multivariable analyses, although the CI was wide (OR, 1.11; CI, 0.92–1.34) None of the remaining findings in the multivariable analyses among women were replicated (Fig. 2). We did not find any associations for the remaining CVD risk factors among women nor any associations among men (Fig. 2). ORs per absolute unit increase in the level of CVD risk factors on the raw scale are presented in Supplementary Table S6 for both the multivariable and MR analyses.

### Sensitivity analyses

The inclusion of participants without genotype information in the multivariable analyses and excluding participants with ART pregnancies and restricting to individuals without a registered pregnancy prior to the measurement of the CVD risk factors yielded similar results to the main analyses (Supplementary Figs S1, S2, and S3, respectively). When we examined history of infertility among nulliparous and parous women separately, there was some support of a stronger relationship with both BMI, SBP, DBP, and HDL cholesterol among nulliparous women in the multivariable analyses (Supplementary Fig. S4). When we compared the 2SLS, MR estimates to the estimates from the sensitivity analyses exploring the likelihood of horizontal pleiotropy (IVW, MR-Egger, and median-based regression), we found largely similar estimates across the different approaches (Supplementary Table S7).

## Discussion

In this large population-based cohort study, an increased risk of infertility was observed among women with higher BMI, lower levels of HDL cholesterol, higher levels of triglycerides, higher lifetime smoking index, higher smoking intensity and among smokers compared to non-smokers using conventional linear multivariable regression. Only the relationship with smoking initiation was significantly associated with infertility in MR analyses. For BMI, the effect estimates were similar using both methods. Among men, there was a decreased risk of infertility with higher DBP and LDL cholesterol in conventional linear multivariable regression, but none of these associations were replicated using MR. We were somewhat underpowered to properly investigate potential non-linear effects.

Our findings in the conventional multivariable analyses are in line with previous studies indicating associations of BMI, lipid profiles, and smoking with infertility among women (Deyhoul *et al.*, 2017; Moridi *et al.*, 2019; Hernáez *et al.*, 2021). There are indeed plausible explanations for a role of these CVD risk factors in infertility. Lipoprotein levels are correlated with hormone levels, including leptin and insulin, which in turn impact reproduction (Rainwater *et al.*, 1997; Mantizoros, 2000; O’Rourke *et al.*, 2001). Leptin, for example, impacts both LH and oestradiol levels (Mantizoros, 2000; Lundåsen *et al.*, 2003; Metwally *et al.*, 2008; Martins *et al.*, 2012). BMI is also associated with oxidative stress, which is linked to female infertility (Ruder *et al.*, 2009). Finally, compounds found in smoke may affect ovarian follicle maturation, ovum differentiation, steroid hormone production, and uterine blood flow, which may all impact the likelihood of infertility (Baird and Wilcox, 1985; Hyland *et al.*, 2016). It is also possible that the association between smoking and infertility might partly reflect maternal smoking, as prenatal exposure to smoking could impact female risk of infertility, and offspring of mothers who smoked might also be more likely to smoke themselves (Weinberg *et al.*, 1989; Wesselink *et al.*, 2018).

We are not aware of previous studies indicating a potential decreased risk of infertility among men with higher DBP and LDL cholesterol, nor any potential mechanisms which might explain this. However, it is important to note that the lack of questioning about male infertility in the questionnaires could lead to potential misclassification of infertility among men, as highlighted in our previous study in HUNT (Skåra *et al.*, 2022).

The results from the MR analyses do not appear to be consistent with similar analyses conducted in the MoBa cohort, where we have previously published evidence of a relationship between BMI and infertility among both men and women but no evidence of a relationship with smoking-related traits (Hernáez *et al.*, 2021, 2022). Potential explanations for the discrepancy in the findings for smoking include a potential selection bias in MoBa, as smokers were highly underrepresented in the cohort (Nilsen *et al.*, 2009). Additionally, the reduction in the proportion of smoking in the general population over time could also contribute to these differences (Nilsen *et al.*, 2009; Krokstad *et al.*, 2013; Åsvold *et al.*, 2023). The discrepancy in the findings for BMI might be explained by MoBa having pre-pregnancy measures in both men and women, which are likely to be more reflective of the levels before the couple was trying to conceive, and by the greater power in the MoBa analyses. However, it is important to note that MR analyses may not fully capture the extremes of variation in genetically predicted risk factors, which could be the case for our analyses in HUNT. For example, the range of genetically predicted BMI in HUNT was limited, between 20.2 and 30.2 kg/m^2^, whereas we were able to capture a wider variation in MoBa.

Our findings were largely consistent across sensitivity analyses. Our sensitivity analysis restricting the multivariable analyses to genotyped individuals yielded similar results to the main analyses, indicating no evidence of selection bias in the MR analyses. Moreover, couples undergoing ART are often recommended to make certain lifestyle changes to optimize the chances of conceiving, which could impact our results, but excluding these individuals gave similar results to the main findings. The results from the sensitivity analysis restricted to individuals without any pregnancies registered prior to collection of information on CVD risk factors were also similar to the findings from the main analyses. This indicates that reverse causation is most likely not an issue. We found strengthened results when examining history of infertility among nulliparous women (no registered pregnancies) compared to the main findings, but not when examining history of infertility among parous women (at least one registered pregnancy). As the group of nulliparous women with infertility could represent women with more severe infertility, this may indicate that any effects of the CVD risk factors could be stronger for women with severe infertility. However, we were likely underpowered for the evaluation of nulliparous women with a history of infertility in MR analyses, and larger studies with more information on the severity of the infertility are warranted to further investigate this.

History of infertility (prolonged time-to-pregnancy) is a couple-level phenotype. There are many different contributing factors to infertility, including hormonal imbalances, infections, and cancer or cancer treatments in both women and men, oocyte quality and quantity, endometriosis, and polycystic ovary syndrome in women, and varicocele and various sperm parameters in men (Healy *et al.*, 1994; Smith *et al.*, 2003; Al Awlaqi *et al.*, 2016; Machen and Sandlow, 2020). Detailed information on these contributing factors was not available in HUNT. It is therefore possible that the CVD risk factors of interest might impact specific causes of infertility, but we were unable to test this. This could potentially be further explored in future studies with this information available.

As mentioned, MR results can be confounded by weak genetic instruments, population structure, and horizontal pleiotropy (Greenland, 2000; Davey Smith and Ebrahim, 2003; Smith and Ebrahim, 2004; Hemani *et al.*, 2018). The genetic instruments for all CVD risk factors were robust in our analyses, except for the three smoking-related traits. This could have resulted in an underestimation of the association between these traits and infertility (Mounier and Kutalik, 2023). The high homogeneity of the HUNT population and our adjustment for principal components reduced any impact on MR results caused by population structure. According to the different MR methods with various assumptions testing for horizontal pleiotropy, we found similar effect sizes using these methods to the main analyses, suggesting minimal evidence of horizonal pleiotropy.

This study has notable strengths, including the large sample size, the homogeneous population, the opportunity to include women who never conceived, and the evaluation of a broad range of CVD risk factors. Despite its contributions, our study also has some limitations worth noting. We were not able to include nulliparous men in our study population, which could have diluted any potential effects among men. Moreover, we did not have information on causes of infertility, so we were only able to evaluate overall history of infertility at the couple level. However, as specific underlying causes of infertility are often not identified, we strongly believe that there is value to the evaluation of this heterogeneous outcome, as it provides the opportunity to explore a potential role of important modifiable risk factors. This could improve future treatment strategies for couples with infertility, especially the ones with unexplained infertility. Moreover, it is important to acknowledge that the lipid measurements were taken from non-fasting participants. The CVD risk factors could also be highly time-specific, and our current data might not adequately reflect the exposure levels during the period that the couples were trying to conceive. However, CVD risk factors often track across the life course (Cheng *et al.*, 2012; Aarestrup *et al.*, 2016). Nonetheless, we attempted to minimize this bias by restricting to individuals who had CVD risk factor measurements at 45 years or younger, and for whom this information was collected prior to or at the same time as the information on infertility. Finally, another limitation arises from the potential impact of shared environmental factors or assortative mating, which could introduce bias to our multivariable and MR analyses (Silventoinen *et al.*, 2003; Clarke *et al.*, 2021). Familial effects could also bias our results, as we were not able identify relatives and conduct within-family analyses (Brumpton *et al.*, 2020).

In conclusion, our study revealed evidence to support a relationship between smoking initiation and history of infertility among women, supported by both conventional multivariable and MR analyses. However, additional MR studies of the relationship between CVD risk factors and infertility with more information on infertility history, especially among men, would be of value.

## Supplementary Material

hoae033_Supplementary_Data

## Data Availability

Participants in The HUNT Study signed an informed consent form allowing the use of their data and samples for research. This does not allow for storage of data on an individual level, in repositories or journals. Participants are not identifiable for researchers using HUNT data. Researchers who want access to HUNT material for replication should apply to HUNT’s Data Access Committee. Access to datasets requires approval from the Regional Committee for Medical and Health Research Ethics in Norway and an agreement with HUNT.
