# Supplementary material for: Cardiovascular disease risk factors and infertility: multivariable analyses and one-sample Mendelian randomization analyses in the Trøndelag Health Study
Source: Hum Reprod Open. 2024 May 24;2024(3):hoae033. doi: 10.1093/hropen/hoae033 (PMC11190059; doi:10.1093/hropen/hoae033)
Supplement: hoae033_Supplementary_Data [file hoae033_supplementary_data.docx]

**Supplementary Data**

**Supplementary Table S1.** Information on the single-nucleotide polymorphisms included in the genetic risk scores for each cardiovascular disease risk factor.

| **Exposures** | **GWAS** | **SNPs in GWAS** | **SNPs in HUNT** |
| --- | --- | --- | --- |
| BMI | Yengo, Sidorenko (1) | 941 | 939 |
| Diastolic BP | Evangelou, Warren (2) | 883 | 856 |
| Systolic BP | Evangelou, Warren (2) | 883 | 856 |
| HDL cholesterol | Graham, Clarke (3) | 380 | 362 |
| Triglycerides | Graham, Clarke (3) | 403 | 376 |
| LDL cholesterol | Graham, Clarke (3) | 403 | 375 |
| Lifetime smoking index | Wootton, Richmond (4) | 126 | 126 |
| Smoking intensity | Liu, Jiang (5) | 55 | 55 |
| Smoking initiation | Liu, Jiang (5) | 378 | 376 |

BP, blood pressure; GWAS, genome-wide association study; HDL, high-density lipoprotein; HUNT, The Trøndelag Health Study; LDL, low-density lipoprotein; SNP, single-nucleotide polymorphism.

**Supplementary Table S2.** Age distribution at the time of measurement of cardiovascular disease risk factors by fertility history among women and men.

| **Exposures** | **Fertile**  **Median (IQR)** | **Infertile**  **Median (IQR)** |
| --- | --- | --- |
| **Women** | | |
| BMI | 34.9 (28.8, 40.1) | 33.9 (28.0, 39.2) |
| Diastolic BP | 34.9 (28.8, 40.1) | 33.9 (27.9, 39.2) |
| Systolic BP | 34.9 (28.8, 40.1) | 33.9 (27.9, 39.2) |
| HDL cholesterol | 34.9 (28.8, 40.1) | 33.9 (28.0, 39.2) |
| Triglycerides | 34.9 (28.8, 40.1) | 33.9 (28.0, 39.2) |
| LDL cholesterol | 34.9 (28.8, 40.1) | 33.8 (28.0, 39.2) |
| **Men** | | |
| BMI | 36.1 (30.1, 40.8) | 34.8 (29.3, 39.9) |
| Diastolic BP | 36.1 (30.1, 40.9) | 34.8 (29.3, 39.9) |
| Systolic BP | 36.1 (30.1, 40.9) | 34.8 (29.3, 39.9) |
| HDL cholesterol | 36.1 (30.1, 40.9) | 34.8 (29.3, 39.8) |
| Triglycerides | 36.1 (30.1, 40.8) | 34.7 (29.3, 39.9) |
| LDL cholesterol | 36.1 (30.1, 40.8) | 34.8 (29.3, 39.9) |

BP, blood pressure; HDL, high-density lipoprotein; LDL, low-density lipoprotein.

**Supplementary Table S3.** Distribution of exposures among eligible women and men for the analyses of each cardiovascular disease risk factor.

| **Exposures** | **Units** | **Fertile**  **Median (IQR)** | **Fertile**  **No.** | **Infertile**  **Median (IQR)** | **Infertile**  **No.** |
| --- | --- | --- | --- | --- | --- |
| **Women** | | | | | |
| BMI | kg/m^2^ | 24.3 (22.2, 27.2) | 11,229 | 24.7 (22.3, 28.0) | 3,123 |
| Diastolic BP | mmHg | 71.0 (65.0, 78.0) | 11,262 | 71.0 (65.0, 78.0) | 3,127 |
| Systolic BP | mmHg | 119.0 (112.0, 128.0) | 11,262 | 119.0 (112.0, 128.0) | 3,127 |
| HDL cholesterol | mg/dL | 54.1 (46.4, 65.7) | 11,225 | 54.1 (46.4, 65.7) | 3,110 |
| Triglycerides | log mg/dL | 93.0 (69.1, 132.9) | 11,259 | 97.4 (70.0, 141.7) | 3,125 |
| LDL cholesterol | mg/dL | 117.9 (97.5, 141.3) | 11,216 | 117.5 (97.8, 142.8) | 3,098 |
| Lifetime smoking index^a,b^ | No unit | 0.2 (0.0, 1.2) | 20,827 | 0.4 (0.0, 1.3) | 4,473 |
| Smoking intensity^c^ | Cigarettes per day | 10 (7, 15) | 11,217 | 10 (8, 15) | 2,591 |
| Smoking initiation^d^ | Yes/no | Yes=12,399; no=9,683 | 22,082 | Yes=2,803; no=1,886 | 4,689 |
| **Men** | | | | | |
| BMI | kg/m^2^ | 25.7 (23.8, 27.9) | 6,181 | 25.9 (23.9, 28.1) | 1,583 |
| Diastolic BP | mmHg | 76.0 (69.0, 82.0) | 6,181 | 76.0 (69.0, 82.0) | 1,582 |
| Systolic BP | mmHg | 131.0 (123.0, 140.0) | 6,181 | 131.0 (123.0, 139.0) | 1,582 |
| HDL cholesterol | mg/dL | 46.4 (38.7, 54.1) | 6,160 | 46.4 (38.7, 54.1) | 1,575 |
| Triglycerides | log mg/dL | 137.3 (96.5, 201.9) | 6,177 | 140.8 (96.5, 203.7) | 1,581 |
| LDL cholesterol | mg/dL | 130.1 (105.9, 154.7) | 6,148 | 127.2 (104.7, 152.4) | 1,569 |
| Lifetime smoking index^a,b^ | No unit | 0.4 (0.0, 1.5) | 12,302 | 0.2 (0.0, 1.3) | 2,382 |
| Smoking intensity^c^ | Cigarettes per day | 15 (10, 20) | 6,959 | 13 (10, 20) | 1,286 |
| Smoking initiation^d^ | Yes/no | Yes=7,589; no=5,475 | 13,064 | Yes=1,404; no=1,099 | 2,503 |

BP, blood pressure; HDL, high-density lipoprotein; LDL, low-density lipoprotein.

^a^ Lifetime smoking index: Integrating smoking duration, heaviness, and cessation for both daily and occasional smokers.

^b^ Indexes are unitless measures.

^c^ Includes only current and former daily smokers.

^d^ Compares current and former daily smokers to non-smokers.

**Supplementary Table S4.** Tests for non-linear associations in multivariable analyses.

|  | **Multivariable linear models** | | **Multivariable non-linear models** | | |
| --- | --- | --- | --- | --- | --- |
| **Exposures** | **AIC** | **P value** | **AIC** | **EDF^c^** | **P value** |
| **Women** |  |  |  |  |  |
| BMI | 14,542 | <0.05 | 14,521 | 3.6 | <0.05 |
| Diastolic BP | 14,611 | 0.09 | 14,611 | 1.0 | 0.09 |
| Systolic BP | 14,614 | 0.90 | 14,613 | 1.8 | 0.40 |
| HDL cholesterol | 14,535 | <0.05 | 14,527 | 2.9 | <0.05 |
| Triglycerides | 14,584 | <0.05 | 14,580 | 4.1 | <0.05 |
| LDL cholesterol | 14,512 | 0.57 | 14,512 | 1.0 | 0.57 |
| Lifetime smoking index^a^ | 22,956 | <0.05 | 22,940 | 4.2 | <0.05 |
| Smoking intensity^b^ | 13,009 | <0.05 | 13,004 | 2.7 | <0.05 |
| **Men** |  |  |  |  |  |
| BMI | 7,688 | 0.15 | 7,688 | 1.0 | 0.15 |
| Diastolic BP | 7,681 | 0.02 | 7,681 | 1.0 | 0.02 |
| Systolic BP | 7,687 | 0.72 | 7,687 | 1.0 | 0.73 |
| HDL cholesterol | 7,652 | 0.05 | 7,652 | 1.0 | 0.05 |
| Triglycerides | 7,680 | 0.13 | 7,680 | 1.0 | 0.13 |
| LDL cholesterol | 7,623 | <0.05 | 7,623 | 1.0 | <0.05 |
| Lifetime smoking index^a^ | 12,802 | 0.29 | 12,802 | 1.0 | 0.29 |
| Smoking intensity^b^ | 7,010 | 0.69 | 7,010 | 1.0 | 0.69 |

AIC, Akaike information criterion; BP, blood pressure; EDF, effective degrees of freedom; HDL, high-density lipoprotein; LDL, low-density lipoprotein.

^a^ Lifetime smoking index: Integrating smoking duration. heaviness. and cessation for both daily and occasional smokers. Also includes non-smokers.

^b^ Includes only current and former daily smokers.

^c^ An EDF of 1.0 implies a linear association.

**Supplementary Table S5.** Robustness of genetic risk scores for the cardiovascular risk factors investigated.

| **Exposures** | **Units** | **Mean** | **SD** | **No.** | **Estimate** | **LCI** | **UCI** | **F-statistic^e^** | **P value** | **Adjusted R^2e^** |
| --- | --- | --- | --- | --- | --- | --- | --- | --- | --- | --- |
| **Women** | | | | | | | | | | |
| BMI | kg/m^2^ | 893.0 | 17.5 | 939 | 0.05 | 0.05 | 0.06 | 680.7 | <0.05 | 0.045 |
| Diastolic BP | mmHg | 848.0 | 18.0 | 856 | 0.08 | 0.07 | 0.08 | 272.7 | <0.05 | 0.018 |
| Systolic BP | mmHg | 848.0 | 18.0 | 856 | 0.12 | 0.11 | 0.13 | 371.8 | <0.05 | 0.025 |
| HDL cholesterol | mg/dL | 399.5 | 8.2 | 362 | 0.59 | 0.56 | 0.61 | 2,077.6 | <0.05 | 0.126 |
| Triglycerides | log mg/dL | 442.6 | 8.3 | 376 | 2.16 | 2.03 | 2.28 | 1,121.6 | <0.05 | 0.072 |
| LDL cholesterol | mg/dL | 402.9 | 7.9 | 375 | 1.80 | 1.73 | 1.86 | 3,004.2 | <0.05 | 0.173 |
| Lifetime smoking index^a,b^ | No unit | 118.8 | 6.8 | 126 | 0.01 | 0.01 | 0.01 | 233.7 | <0.05 | 0.009 |
| Smoking intensity^c^ | Cigarettes per day | 61.6 | 6.7 | 55 | 0.09 | 0.08 | 0.11 | 142.1 | <0.05 | 0.010 |
| Smoking initiation^d^ | Yes/no | 398.4 | 11.9 | 376 | 0.03 | 0.02 | 0.03 | 0.5 | <0.05 | 0.029 |
| **Men** | | | | | | | | | | |
| BMI | kg/m^2^ | 892.8 | 17.4 | 939 | 0.04 | 0.03 | 0.04 | 310.7 | <0.05 | 0.038 |
| Diastolic BP | mmHg | 847.4 | 18.0 | 856 | 0.08 | 0.07 | 0.10 | 170.0 | <0.05 | 0.022 |
| Systolic BP | mmHg | 847.4 | 18.0 | 856 | 0.13 | 0.11 | 0.14 | 242.8 | <0.05 | 0.031 |
| HDL cholesterol | mg/dL | 399.3 | 8.2 | 380 | 0.53 | 0.50 | 0.56 | 1,246.7 | <0.05 | 0.139 |
| Triglycerides | log mg/dL | 442.5 | 8.3 | 403 | 3.50 | 3.20 | 3.76 | 595.3 | <0.05 | 0.072 |
| LDL cholesterol | mg/dL | 402.7 | 7.9 | 403 | 1.87 | 1.78 | 1.96 | 1,507.2 | <0.05 | 0.165 |
| Lifetime smoking index^a,b^ | No unit | 118.6 | 6.8 | 126 | 0.01 | 0.01 | 0.01 | 95.0 | <0.05 | 0.007 |
| Smoking intensity^c^ | Cigarettes per day | 61.5 | 6.7 | 55 | 0.09 | 0.07 | 0.12 | 47.8 | <0.05 | 0.005 |
| Smoking initiation^d^ | Yes/no | 398.1 | 12.0 | 376 | 0.03 | 0.02 | 0.03 | 0.5 | <0.05 | 0.028 |

BP, blood pressure; HDL, high-density lipoprotein; LCI, 95% lower confidence interval; LDL, low-density lipoprotein; UCI, 95% upper confidence interval.

^a^ Lifetime smoking index: Integrating smoking duration, heaviness, and cessation for both daily and occasional smokers.

^b^ Indexes are unitless measures.

^c^ Includes only current and former daily smokers.

^d^ Compares current and former daily smokers to non-smokers.

^e^ Partial area under the receiver operating characteristics (ROC) curve and partial pseudo- R^2^ using the Nagelkerke method were calculated for smoking initiation.

**Supplementary Table S6.** Risk of infertility per unit increase in the levels of cardiovascular risk factors for women and men.

| **Exposures** | **Units** | **Mean (SD)** | **Multivariable analyses**  **OR (CI)** | **Mendelian randomization analyses**  **OR (CI)** |
| --- | --- | --- | --- | --- |
| **Women** | | | | |
| BMI | kg/m^2^ | 25.3 (4.4) | 1.03 (1.02, 1.04) | 1.03 (0.98, 1.07) |
| Diastolic BP | mmHg | 72.0 (10.1) | 1.00 (0.99, 1.00) | 1.00 (0.97, 1.03) |
| Systolic BP | mmHg | 120.8 (13.4) | 1.00 (1.00, 1.00) | 1.00 (0.98, 1.02) |
| HDL cholesterol | mg/dL | 56.6 (13.5) | 0.99 (0.99, 1.00) | 1.00 (0.99, 1.01) |
| Triglycerides | log mg/dL | 111.8 (66.4) | 1.00 (1.00, 1.00) | 1.00 (1.00, 1.00) |
| LDL cholesterol | mg/dL | 121.5 (34.4) | 1.00 (1.00, 1.00) | 1.00 (1.00, 1.00) |
| Lifetime smoking index^a,b^ | No unit | 0.6 (0.7) | 1.24 (1.18, 1.30) | 1.55 (0.94, 2.55) |
| Smoking intensity^c^ | Cigarettes per day | 11.3 (6.2) | 1.02 (1.02, 1.03) | 1.02 (0.95, 1.09) |
| Smoking initiation^d,e^ | Yes/no | NA | 1.22 (1.14, 1.31) | 1.12 (1.01, 1.25) |
| **Men** | | | | |
| BMI | kg/m^2^ | 26.1 (3.3) | 1.01 (1.00, 1.03) | 1.03 (0.95, 1.12) |
| Diastolic BP | mmHg | 76.3 (10.3) | 0.99 (0.99, 1.00) | 1.00 (0.96, 1.04) |
| Systolic BP | mmHg | 131.9 (13.0) | 1.00 (0.99, 1.00) | 1.00 (0.97, 1.02) |
| HDL cholesterol | mg/dL | 46.8 (11.5) | 0.99 (0.99, 1.00) | 0.99 (0.98, 1.01) |
| Triglycerides | log mg/dL | 165.1 (107.6) | 1.00 (1.00, 1.00) | 1.00 (1.00, 1.00) |
| LDL cholesterol | mg/dL | 131.4 (36.8) | 1.00 (1.00, 1.00) | 1.00 (0.99, 1.00) |
| Lifetime smoking index^a,b^ | No unit | 0.7 (0.8) | 0.97 (0.91, 1.03) | 1.14 (0.56, 2.33) |
| Smoking intensity^c^ | Cigarettes per day | 14.9 (8.5) | 1.00 (0.99, 1.01) | 1.06 (0.96, 1.17) |
| Smoking initiation^d,e^ | Yes/no | NA | 1.02 (0.93, 1.11) | 0.99 (0.86, 1.15) |

BP, blood pressure; HDL, high-density lipoprotein; LDL, low-density lipoprotein; NA, not available; OR, odds ratio

^a^ Lifetime smoking index: Integrating smoking duration, heaviness, and cessation for both daily and occasional smokers.

^b^ Indexes are unitless measures.

^c^ Includes only current and former daily smokers.

^d^ Compares current and former daily smokers to non-smokers.

^e^ Binary variable, mean not available.

**Supplementary Table S7.** Risk of infertility per unit increase in the levels of cardiovascular disease risk factors for main analyses and sensitivity analyses in Mendelian randomization.

| **Exposures** | **Units** | **TSPS**  **OR (CI)** | **IVW**  **OR (CI)** | **MR-Egger**  **OR (CI)** | **Weighted median**  **OR (CI)** |
| --- | --- | --- | --- | --- | --- |
| **Women** | | | | | |
| BMI | kg/m^2^ | 1.03 (0.98, 1.07) | 1.03 (1.00, 1.05) | 1.02 (0.99, 1.05) | 1.02 (0.98, 1.06) |
| Diastolic BP | mmHg | 1.00 (0.97, 1.03) | 0.99 (0.96, 1.02) | 0.96 (0.91, 1.01) | 0.98 (0.93, 1.03) |
| Systolic BP | mmHg | 1.00 (0.98, 1.02) | 0.99 (0.96, 1.02) | 0.96 (0.91, 1.01) | 0.98 (0.93, 1.03) |
| HDL cholesterol | mg/dL | 1.00 (0.99, 1.01) | 0.94 (0.45, 1.96) | 1.03 (0.29, 3.68) | 1.15 (0.44, 2.98) |
| Triglycerides | log mg/dL | 1.00 (1.00, 1.00) | 1.00 (1.00, 1.00) | 1.00 (1.00, 1.01) | 1.00 (1.00, 1.00) |
| LDL cholesterol | mg/dL | 1.00 (1.00, 1.00) | 1.00 (1.00, 1.00) | 1.00 (0.99, 1.01) | 1.00 (1.00, 1.00) |
| Lifetime smoking index^a^ | No unit | 1.55 (0.94, 2.55) | 1.39 (0.93, 2.07) | 1.50 (0.70, 3.24) | 1.31 (0.73, 2.35) |
| Smoking intensity^b^ | Cigarettes per day | 1.02 (0.95, 1.09) | 1.04 (1.01, 1.08) | 1.08 (1.03, 1.13) | 1.06 (1.01, 1.12) |
| Smoking initiation^c^ | Yes/no | 1.12 (1.01, 1.25) | 1.09 (1.01, 1.18) | 1.15 (0.98, 1.34) | 1.11 (0.98, 1.25) |
| **Men** | | | | | |
| BMI | kg/m^2^ | 1.03 (0.95, 1.12) | 1.02 (0.98, 1.06) | 1.02 (0.97, 1.07) | 1.04 (0.98, 1.10) |
| Diastolic BP | mmHg | 1.00 (0.96, 1.04) | 1.01 (0.99, 1.02) | 1.01 (0.99, 1.03) | 1.00 (0.98, 1.02) |
| Systolic BP | mmHg | 1.00 (0.97, 1.02) | 1.00 (0.99, 1.01) | 1.01 (0.99, 1.03) | 1.01 (0.99, 1.03) |
| HDL cholesterol | mg/dL | 0.99 (0.98, 1.01) | 0.99 (0.98, 1.00) | 0.99 (0.96, 1.01) | 0.98 (0.96, 1.00) |
| Triglycerides | log mg/dL | 1.00 (1.00, 1.00) | 1.00 (1.00, 1.00) | 1.00 (1.00, 1.00) | 1.00 (1.00, 1.00) |
| LDL cholesterol | mg/dL | 1.00 (0.99, 1.00) | 1.00 (1.00, 1.00) | 1.00 (0.99, 1.01) | 1.00 (0.99, 1.00) |
| Lifetime smoking index^a^ | No unit | 1.14 (0.56, 2.33) | 1.19 (0.76, 1.86) | 1.69 (0.72, 4.01) | 0.91 (0.47, 1.74) |
| Smoking intensity^b^ | Cigarettes per day | 1.06 (0.96, 1.17) | 1.03 (0.96, 1.12) | 0.97 (0.85, 1.10) | 1.09 (1.01, 1.18) |
| Smoking initiation^c^ | Yes/no | 0.99 (0.86, 1.15) | 1.02 (0.93, 1.12) | 1.10 (0.93, 1.31) | 1.07 (0.92, 1.23) |

BP, blood pressure; CI, HDL, high-density lipoprotein; IVW, inverse-variance weighted analysis; LDL, low-density lipoprotein; OR, odds ratio; TSPS, two-stage predictor substitution.

^a^ Lifetime smoking index: Integrating smoking duration. heaviness. and cessation for both daily and occasional smokers.

^b^ Includes only current and former daily smokers.

^c^ Compares current and former daily smokers to non-smokers.


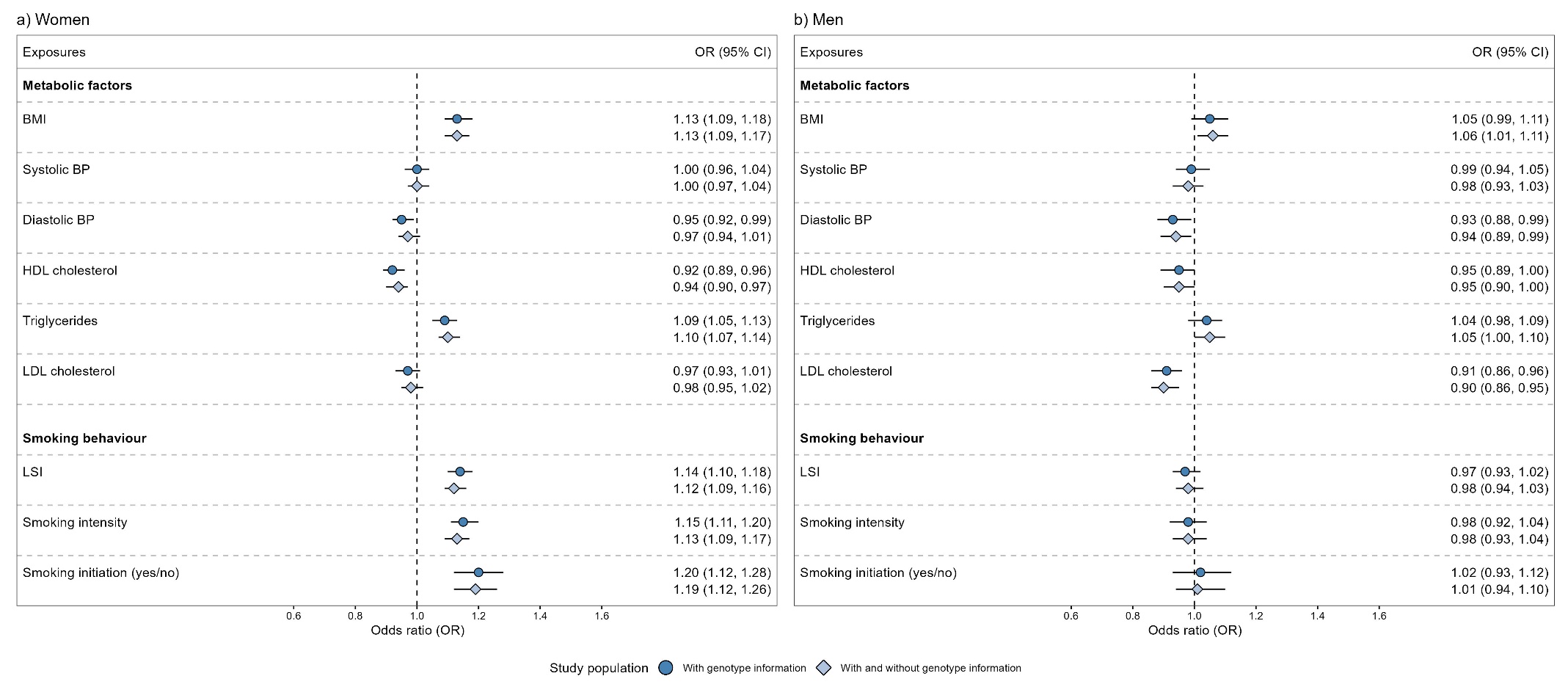


**Supplementary Figure S1.** Associations between cardiovascular disease risk factors and infertility in a) women and b) men with and without genotype information.

BP, blood pressure; HDL, high-density lipoprotein; LDL, low-density lipoprotein, LSI, lifetime smoking index.


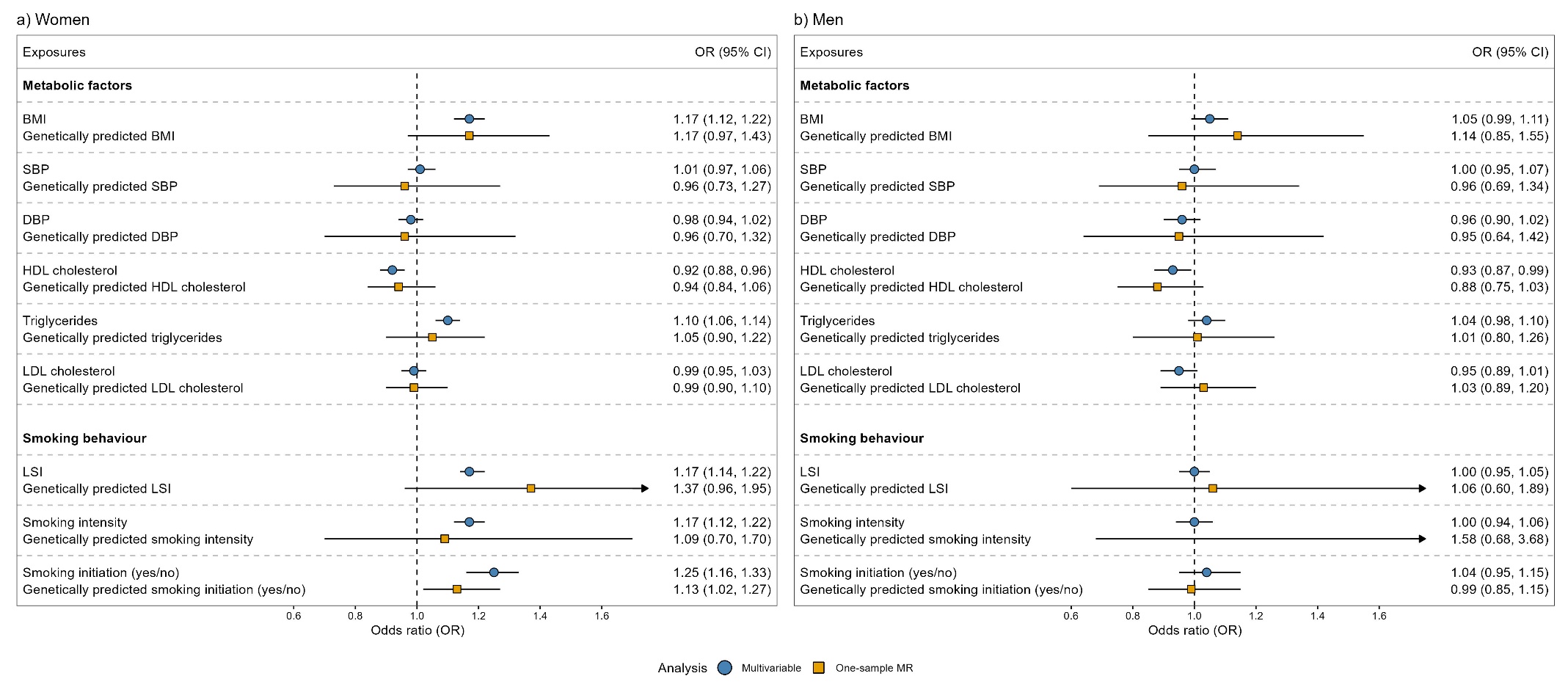


**Supplementary Figure S2.** Associations between cardiovascular disease risk factors and infertility in a) women and b) men excluding participants using assisted reproductive technologies to conceive.

BP, blood pressure; HDL, high-density lipoprotein; LDL, low-density lipoprotein, LSI, lifetime smoking index.


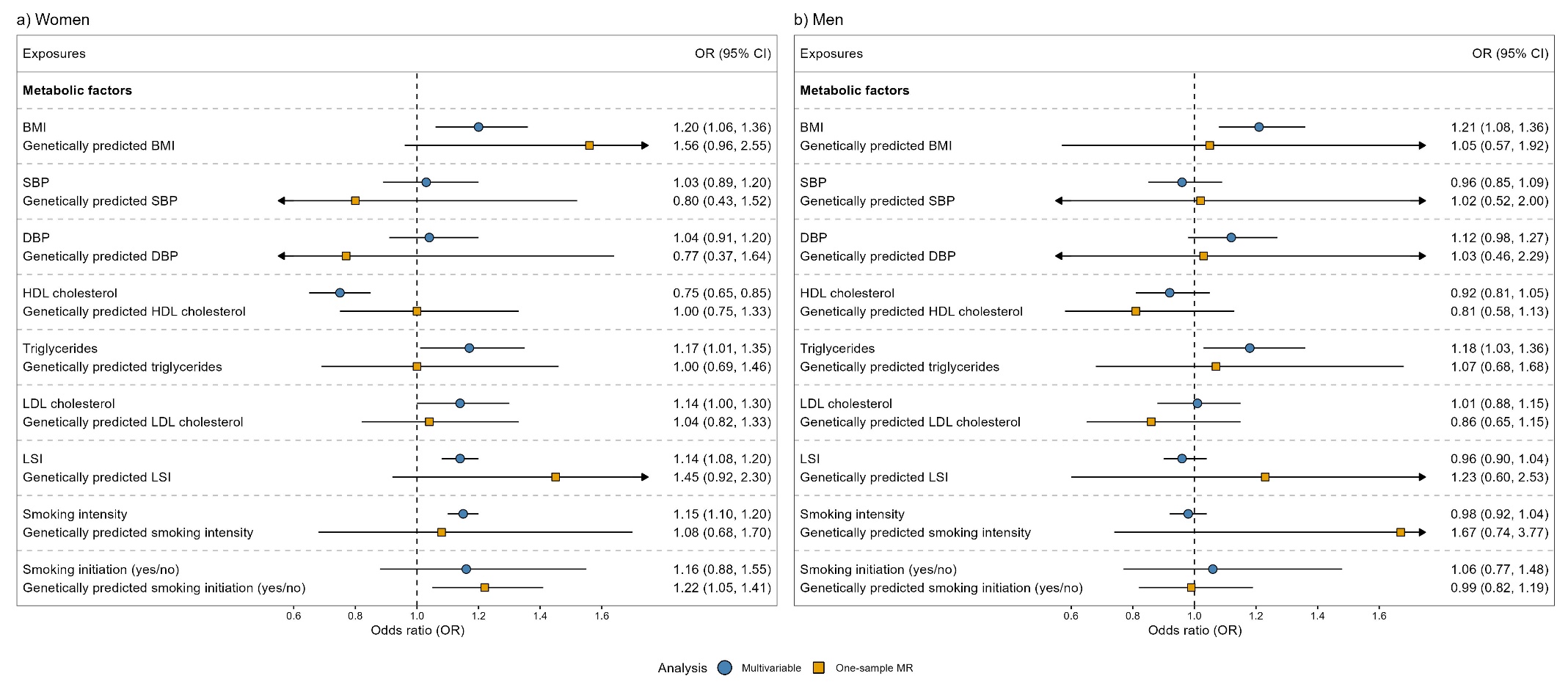


**Supplementary Figure S3.** Associations between cardiovascular disease risk factors and infertility in a) women and b) men excluding participants with pregnancies registered prior to cardiovascular disease risk factor measurements or prior to smoking initiation.

BP, blood pressure; HDL, high-density lipoprotein; LDL, low-density lipoprotein, LSI, lifetime smoking index.


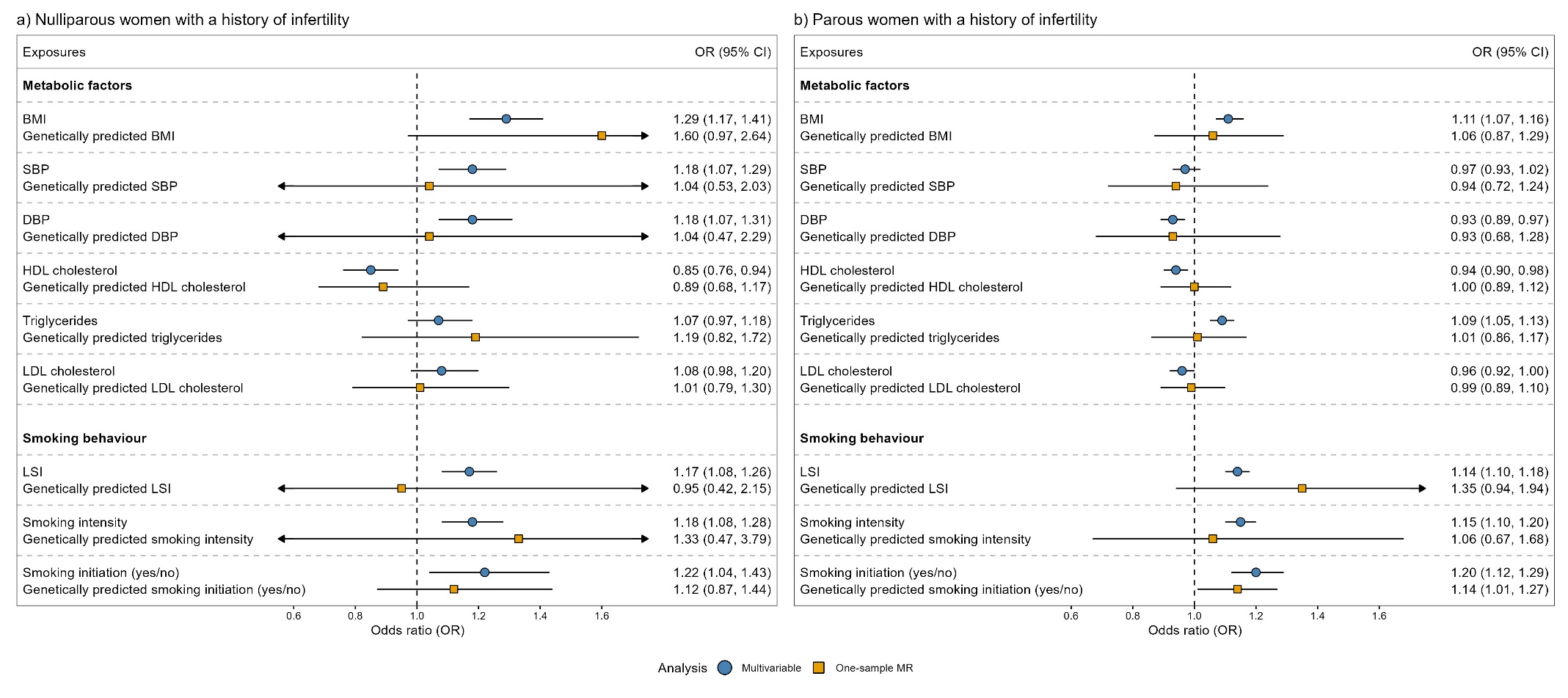


**Supplementary Figure S4.** Associations between cardiovascular disease risk factors and infertility in a) nulliparous women with infertility (no registered pregnancies) compared to fertile women and b) parous women with infertility (at least one registered pregnancy) compared to fertile women.

BP, blood pressure; HDL, high-density lipoprotein; LDL, low-density lipoprotein, LSI, lifetime smoking index.

**References**

Yengo L, Sidorenko J, Kemper KE, Zheng Z, Wood AR, Weedon MN, et al. Meta-analysis of genome-wide association studies for height and body mass index in ∼700000 individuals of European ancestry. Hum Mol Genet. 2018;27(20):3641-9.

Evangelou E, Warren HR, Mosen-Ansorena D, Mifsud B, Pazoki R, Gao H, et al. Genetic analysis of over 1 million people identifies 535 new loci associated with blood pressure traits. Nat Genet. 2018;50(10):1412-25.

Graham SE, Clarke SL, Wu K-HH, Kanoni S, Zajac GJM, Ramdas S, et al. The power of genetic diversity in genome-wide association studies of lipids. Nature. 2021;600(7890):675-9.

Wootton RE, Richmond RC, Stuijfzand BG, Lawn RB, Sallis HM, Taylor GMJ, et al. Evidence for causal effects of lifetime smoking on risk for depression and schizophrenia: a Mendelian randomisation study. Psychol Med. 2020;50(14):2435-43.

Liu M, Jiang Y, Wedow R, Li Y, Brazel DM, Chen F, et al. Association studies of up to 1.2 million individuals yield new insights into the genetic etiology of tobacco and alcohol use. Nat Genet. 2019;51(2):237-44.
